# Enhancer/Promoter Activities of the Long/Middle Wavelength-Sensitive Opsins of Vertebrates Mediated by Thyroid Hormone Receptor β2 and COUP-TFII

**DOI:** 10.1371/journal.pone.0072065

**Published:** 2013-08-23

**Authors:** Toshiro Iwagawa, Yo Tanaka, Atsumi Iida, Toshio Itoh, Sumiko Watanabe

**Affiliations:** 1 Division of Molecular and Developmental Biology, Institute of Medical Science, University of Tokyo, Tokyo, Japan; 2 Marmoset Research Department, Central Institute for Experimental Animals, Kawasaki, Japan; University of Washington, United States of America

## Abstract

Cone photopigments (opsins) are crucial elements of, and the first detection module in, color vision. Individual opsins have different wavelength sensitivity patterns, and the temporal and spatial expression patterns of opsins are unique and stringently regulated. Long and middle wavelength-sensitive (L/M) opsins are of the same phylogenetic type. Although the roles of thyroid hormone/TRß2 and COUP-TFs in the transcriptional regulation of L/M opsins have been explored, the detailed mechanisms, including the target sequence in the enhancer of L/M opsins, have not been revealed. We aimed to reveal molecular mechanisms of L/M opsins in vertebrates. Using several human red opsin enhancer/promoter-luciferase reporter constructs, we found that TRß2 increased luciferase activities through the 5′-UTR and intron 3–4 region, whereas the presence of T3 affected only the intron 3–4 region-dependent luciferase activity. Furthermore, COUP-TFII suppressed intron 3–4 region-dependent luciferase activities. However, luciferase expression driven by the mouse M opsin intron 3–4 region was only slightly increased by TRß2, and rather enhanced by COUP-TFII. To determine whether these differential responses reflect differences between primates and rodents, we examined the enhancer/promoter region of the red opsin of the common marmoset. Interestingly, while TRß2 increased 5′-UTR- or intron 3–4 region-driven luciferase expression, as observed for the human red opsin, expression of the latter luciferase was not suppressed by COUP-TFII. In fact, immunostaining of common marmoset retinal sections revealed expression of COUP-TFII and red opsin in the cone cells.

## Introduction

The retina contains two types of photoreceptors, rods and cones, which are located in the outermost layer of the three-layered retinal structure in vertebrates. Rods are responsible for vision at low light levels and contain rhodopsin, which is activated under scotopic and dark conditions. Cones are color-sensitive, and cone photopigments, known as opsins, are crucial elements in color vision. There are several different opsins, each of which has a distinctive wavelength sensitivity pattern, and the opsin diversification is species-dependent [1]. The acquisition through evolution of species-specific diversity regarding opsin genes in vertebrates is well understood [2]. Vertebrate opsins are classified into five types: RH1 (rod opsins); RH2 (RH1-like [3] cone opsins); SWS1 (short wavelength-sensitive type 2 or [blue] cone opsins); and M/LWS (middle-to-long wavelength-sensitive [red] cone opsins) [4]. Human cone opsins consist of: red opsins (long wavelength); green opsins (middle wavelength); and blue opsins (short wavelength). Comparing the red and green opsins, 349 of the 364 amino acids are identical, and these two genes are arranged in a head-to-tail tandem array on the long arm of the X-chromosome [5], [6]. The mouse has two cone opsins, i.e., M-opsin (middle wavelength opsin) and S-opsin (short wavelength opsin), and the human red opsin is generally considered to correspond to mouse M-opsin based on sequence similarities [2]. The common marmoset possesses a remarkable polymorphism in an X-linked locus, which results in a different spectral sensitivity pattern of the long wavelength opsin [7].

In vertebrates, while the spatial expression pattern of cone opsins across the retina defines the retinal field, the way in which the opsins are arrayed depends on the species. The spatial array of opsins involves species-specific structures, such as the fovea in primates and the visual streak in chickens, and simple dorsal-ventral and nasal-temporal gradients of opsin expression have been observed in fish and rodents [8]–[10]. In mice, the S- and M-opsins have unique and opposite expression patterns in the retina, with gradients along the dorsoventral axis, although cone photoreceptors are distributed throughout the retina [11]. Investigations of the molecular components involved in establishing such spatial expression patterns have shown the involvement of signaling molecules and transcription factors, such as thyroid hormone receptor ß2 (TRß2), retinoid X receptor γ(RXRγ), and retinoid-related orphan receptor ß (RORß) [12]–[14]. We found that BMP and chicken ovalbumin upstream promoter (COUP) transcription factor (TF) family members are crucial to the development of the dorsoventral gradient [15]. BMP signaling is essential for the correct dorsoventral spatial expression of COUP-TFI and COUP-TFII [15]. In addition, COUP-TFI and COUP-TFII are required for the suppression of S-opsin expression in the dorsal retina, whereas only COUP-TFI plays an essential role in the suppression of M-opsin expression in the ventral retina in the mouse [15]. However, the target regions for these transcription factors in the opsin genes have not been fully documented, and whether or not COUP-TFII relies on thyroid hormone receptor ß2 (TRß2) to exert its activities is not known.

Since the expression patterns of human opsins are quite different from those of murine opsins, we investigated whether TRß2 and COUP-TFs regulate the human red opsin gene in a manner similar to that seen for the murine M-opsin.

## Materials and Methods

### Mice, and Common Marmosets

ICR mice were obtained from Japan SLC Co. All animal experiments were approved by the Animal Care Committee of the Institute of Medical Science, University of Tokyo and conducted in accordance with the ARVO (Association for Research in Vision and Ophthalmology) statement for the use of animals in ophthalmic and vision research. Common marmosets (Callithrix jacchus) were purchased from the animal breeding company CLEA Japan and maintained in a cage with dimensions of 50×60×75 cm at Central Institute for Experimental Animals (CIEA). Eyeballs were obtained from marmosets (4 years old) that were sacrificed due to accidents at the CIEA. We confirmed that their eyes were morphological normal by examining sections. Sacrifice of common marmoset was appropriately performed in accordance with CIEA guidelines.

### DNA Construction

Genomic DNA was purified from Y79 retinoblastoma cell line [16], and retinas from mice and marmoset, and construction of reporter plasmids was done using fragments amplified by PCR and restriction enzyme digestion. pGL3 and 4 luciferase vector (Promega) was used for a luciferase vector. To construct mouse COUP-TFII DNA binding domain (DBD) expressing plasmid, a PCR fragment encoding COUP-TFII DBD region (amino acids 79–151) was inserted into the *Eco*RI and *Not*I sites of pcDNA3.1(+) (Life technologies). Constructs containing shRNA anti-COUP-TFI or –TFII were described previously [15]. Primers used for construction are listed in Table S1.

### Cell Culture and Luciferase Analysis

A human embryonic kidney cell line, HEK293T [17] was maintained with DMEM containing 10% of fetal calf serum (FCS, CCT), penicillin (50 units/ml), and streptomycin (50 mg/ml). Human retinoblastoma derived cell line, Y79 cells [16], which were obtained from the Riken Cell Bank (no. RCB1645) was maintained in RPMI1640 (Nacalai tesque) containing 10% FCS (JRH), penicillin, and streptomycin. All cells were cultured in 37 °C, 5% CO2 incubator. Transfection was done using Gene Juice Transfection Reagent (Novagen) according to an instruction supplied with the reagent. Briefly, Gene Juice reagent (1.5 µl) was mixed with 50 µl of Opti-MEM (Life technologies) and incubate 5 minutes, then plasmid (0.5 µg) was mixed and incubate another 15 minutes at room temperature. Cells (HEK293T, 1.7×10^5^ cells) were seed in 12 well plate and cultured overnight, and transfection mixture was added and after 28 hours, cells were harvested. T3 (Sigma-Aldrich) was added at final concentration of 100 nM at 12 hours after transfection. Luciferase activity was analyzed by Luciferase Assay System (Promega) using Lumat LB9507 (Berthold Japan). Data represent the average +/− standard deviation of triplicate samples and are expressed as relative values. Statistical significance was calculated using a 2-tailed student’s T test.

### RT-PCR

HEK293T or Y79 cells (3×10^6^ cells) were seeded in 6 well plate and cultured overnight. Then, plasmid (1 µg) was transfected using Gene Juice Transfection Reagent. After 24 hours of culture, total RNA was extracted by Sepasol RNAI Super G (Nacalai tesque), and cDNA was synthesized using Super ScriptII reverse transcriptase (Life technologies).

### Immunostaining of Common Marmoset Retina

Common marmoset retina was isolated, fixed with 4% PFA and frozen sectioned as previously described [18]. Antibodies used for immunostaining are mouse anti-COUP-TFII (PPMX), rabbit anti-opsin, Red/Green polyclonal (Millipore) antibodies as primary antibodies, and appropriate secondary antibodies conjugated with Alexa-488, or -546. Samples were mounted in VectaShield (Vector Laboratories) and analyzed by using a Zeiss Axio Vision 4.6 microscope.

## Results

### The Human Red Opsin 5′-proximal Region is Activated by TRß2

**Figure 1:**
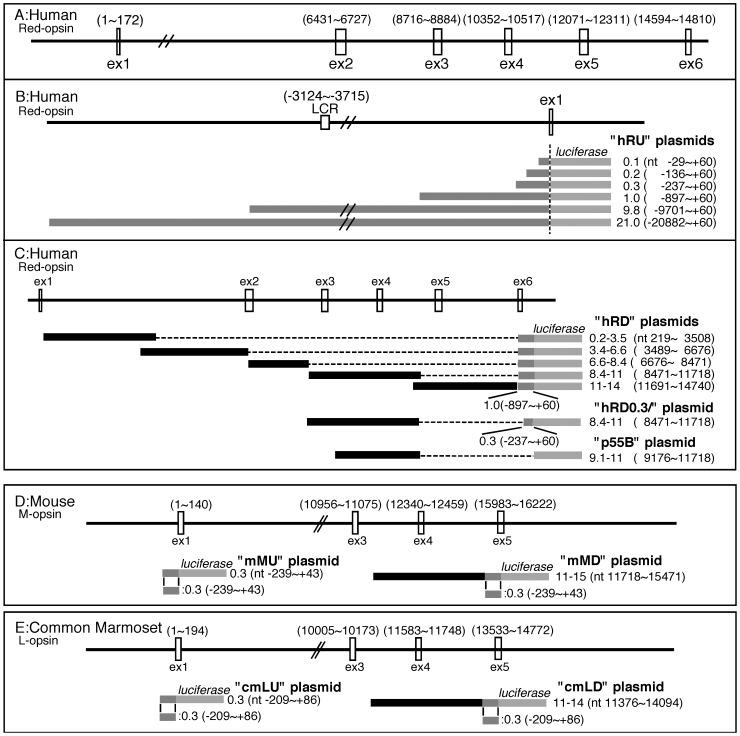
Schematic presentation of opsin promoter-luciferase constructs. A. Exon intron structure of human red opsin gene. B. Luciferase constructs of a serial deletion of 5′ promoter region of human red opsin. C. Luciferase constructs containing various fragments from exon/intron region of human red opsin fused with 1.0 kb, or 0.3 kb of 5′ promoter, or interferon promoter (p55B) region of human red opsin are shown. D. Upper diagram shows exon/intron structure of mouse M-opsin gene. Luciferase constructs of mouse M-opsin promoter are schematically shown in lower part. E. Upper diagram shows exon/intron structure of common marmoset red opsin gene, and luciferase constructs are schematically shown in lower part. Information of exon/intron structure was quoted from Sanger Institute public database, and TSS (transcriptional start site) is designated as nucleotide number 1. nt, nucleotide; ex, exon: LCR, locus control region.

**Figure 2:**
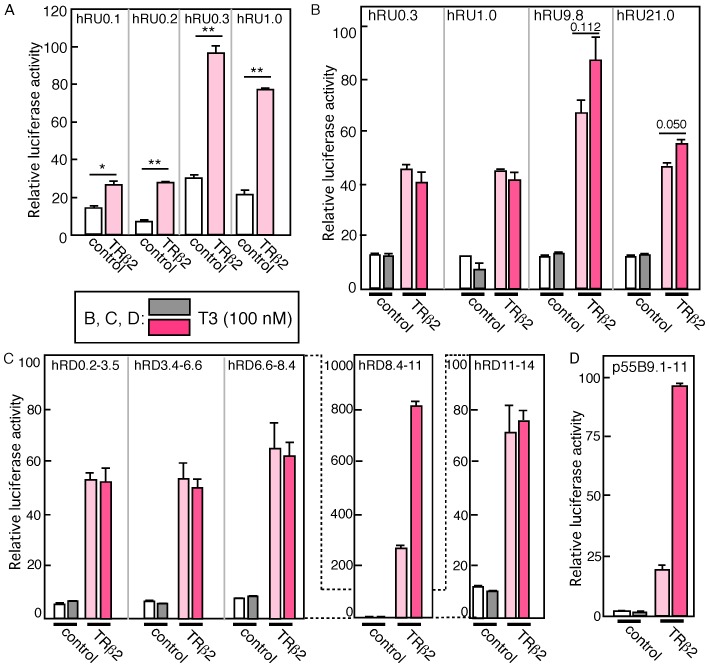
Effects of TRß2 and T3 on human red opsin promoter activities. Luciferase plasmid was transfected into HEK293T cells with TRß2 expression vector or a control vector, and cells were harvested after 28 hours of incubation. Luciferase activities were examined and expressed as relative values. A. Luciferase plasmid containing 5′ proximal promoter region was transfected with TRß2 expression plasmid or control vector. B–D. Luciferase plasmids containing 5′ promoter region (B), exon/intron region fused with 1.0 kb proximal promoter region (C), or p55Bluc vector (D) was transfected into HEK293T with TRß2 expression plasmid or a control vector, and cultured for 12 hours, then cultured additional 16 hours in the presence or absence of T3 (100 nM). Luciferase activity was examined and expressed as relative values. Pink colored bars indicate samples transfected with TRß2, and dark grey and dark pink show those with stimulation by T3. p value; **<0.01, *<0.05, or actual value was calculated by Student’s t-test.

The roles of T3 and TRß in the expression of S-opsin have been elucidated [14], [19], [20]. The addition of T3 was shown to induce the L- and M-opsins in the retinoblastoma cell line WERI [21]. More recently, the involvement of TRß2 in red opsin expression was reported for patients who carry mutations in the thyroid hormone receptor gene [22]. To identify the region of the human red opsin gene (OPN1LW) enhancer that is responsible for thyroid hormone stimulation, we constructed various luciferase reporter plasmids (Fig. 1A, B), and examined the roles of thyroid hormone receptor signals by co-expressing mouse TRß2 in HEK293T cells (Fig. 2A). The luciferase activity of a plasmid that contains a 0.3-kb upstream region was induced by the co-expression of TRß2 (Fig. 2A, hRU0.3). A lower degree of enhancement was observed for a luciferase plasmid that contained either a 0.1-kb or 0.2-kb region (Fig. 2A, hRU0.1, hRU0.2). A plasmid that contained a genomic fragment of 1.0 kb (hRU1.0) also responded to TRß2 expression (Fig. 2A). We then examined the effects of the addition of T3 to the region using the 0.3-kb (hRU0.3) and 1.0-kb (hRU1.0) constructs; no additional luciferase activity was observed (Fig. 2B). We examined the effects of T3 on much longer genomic regions that contained the locus control region (LCR). A luciferase construct that contained a 9.8-kb region (hRU9.8) was induced by TRß2 but did not respond to T3 (Fig. 2B). A plasmid that contained a much longer fragment of ∼21 kb (hRU21) was also induced by TRß2, whereas expression was not enhanced by the addition of T3 (Fig. 2B), which suggests that TRß2 activates the 5′-proximal region of the red opsin gene independently of T3 stimulation.

### The Intron 3–4 Region is Sensitive to TRß2 and T3 Stimulation

We then investigated whether the downstream region contains elements responsible for TRß2/T3 stimulation. We constructed a series of luciferase plasmids that contained fragments covering introns 1 to 5 fused with a 1.0-kb 5′-region of the red opsin promoter (Fig. 1C). The expression levels of luciferase from these plasmids were examined in HEK293T cells (Fig. 2C). The luciferase activities of hRD0.2–3.5, hRD3.4–6.6, and hRD6.6–8.4 were very low, although luciferase activity was enhanced by TRß2 co-expression (Fig. 2C). The luciferase activity of hRD8.4–11 was strongly increased by the co-expression of TRß2, and this activity was further enhanced by the addition of T3 (Fig. 2C). The addition of T3 in the absence of TRß2 did not affect the level of luciferase activity (Fig. 2C). A plasmid that contained a much longer downstream region of the red opsin gene, hRD11–14, showed only weak luciferase activity, and this activity was enhanced by the co-expression of TRß2 but was not further enhanced by the addition of T3 (Fig. 2C). We next examined the enhancer activity of this region in the context of other minimal promoters by subcloning a fragment that contained this region (+9176∼11718) into the p55Bluc vector, which contains the interferon-ß basal promoter (p55B9.1–11). Co-expression of TRß2 increased the luciferase activity of this construct, and the addition of T3 further strongly enhanced this activity (Fig. 2D).

### COUP-TFII Suppresses TRß2-dependent Activation of Red Opsin-luciferase Fusions

**Figure 3:**
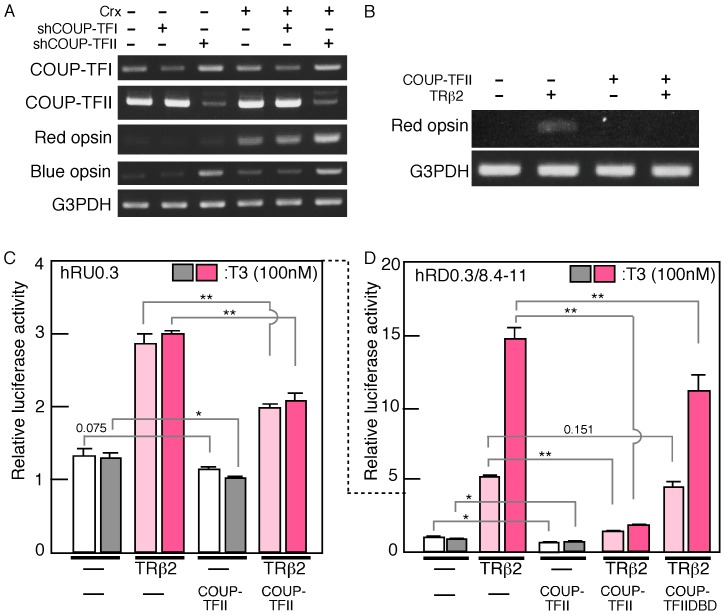
Effects of COUP-TFII for human red opsin promoter activities. A, B. Plasmids encoding U6-sh-COUP-TFI or U6-sh-COUP-TFII with/without Crx expression plasmid were transfected into Y79 (A). Expression plasmid of COUP-TFII and/or TRß2 were transfected to HEK293T (B) cells. Transfected cells were harvested after two days. Then expression of COUP-TFI, COUP-TFII, red opsin, and blue opsin (A), and red opsin (B) was examined by semi-quantitative RT-PCR. G3PDH was used as a control (A, B). C, D. Human red opsin luciferase constructs, hRU0.3 (C) or hRD0.3/8.4–11 (D) was transfected into HEK293T with TRß2 expression plasmid or a control vector, and cultured for 12 hours, T3 (100 nM) was added half of samples and cultured additional 16 hours. p value; **<0.01, *<0.05, or actual value was calculated by Student’s t-test.

We previously reported roles for COUP-TF transcription factor family members in opsin gene expression using the mouse retina [15]. COUP-TFI-knockout mice showed up-regulated expression of both the S- and M-opsins in the retina [15]. Moreover, COUP-TFII-knockout mice showed ectopic expression of S-opsin but no effects on M-opsin expression [15]. These results suggested roles for COUP-TFI in S-opsin and M opsin expression and for COUP-TFII in S-opsin expression. We investigated whether the expression of human red and blue opsins is regulated by COUP-TFs using human Y79 retinoblastoma cells. Initially, we examined the expression levels of the red and blue opsins in the presence of shRNA directed against COUP-TFs. Plasmids that encoded shRNA directed against COUP-TFI or COUP-TFII were transfected into Y79 cells, and the expression levels of COUP-TFI and TF-II and the red and blue opsins were assayed by semi-quantitative RT-PCR (Fig. 3A). Red opsin expression was enhanced by shCOUP-TFII but only weakly by shCOUP-TFI (Fig. 3A). As previously reported [15], the expression of blue opsin was weakly induced by CRX, and this expression level was further increased through suppression of COUP-TFII by shRNA (Fig. 3A). We further confirmed the inhibitory effects of COUP-TFII on red opsin expression in HEK293T cells by semi-quantitative PCR (Fig. 3B). Red opsin expression was induced by the presence of TRß2, and this expression was suppressed by the expression of COUP-TFII (Fig. 3B). We then examined the effects of COUP-TFII using the red opsin luciferase constructs. The hRU0.3 plasmid was co-transfected with TRß2 and/or COUP-TFII into HEK293T cells. Then, half of the samples were stimulated with T3. The luciferase activities of hRU0.3 were strongly suppressed by the co-expression of COUP-TFII but were not affected by the addition of T3 (Fig. 3C), and the basal level of luciferase was not affected by the co-expression of COUP-TFII (Fig. 3C). Then, we constructed a plasmid that contained a 0.3-kb region upstream of the promoter and intron 2–4 region (hRD0.3/8.4–11), and analyzed the luciferase activities in HEK293T cells. The expression of COUP-TFII suppressed TRß2-induced luciferase activity (Fig. 3D). Luciferase activity in the absence of TRß2 was only slightly suppressed by COUP-TFII (Fig. 3D). The presence of T3 further enhanced the luciferase activity, as expected, and this activity was also nearly completely suppressed by the co-expression of COUP-TFII (Fig. 3D). Expression of the COUP-TFII DNA binding domain (COUP-TFII DBD) did not suppress the luciferase activity induced by TRß2 (Fig. 3D), which suggests that COUP-TFII may not simply compete with TRß2 for the target sequence in the red opsin gene.

### Effects of TRß2 and COUP-TFs on Mouse M-opsin Expression

**Figure 4:**
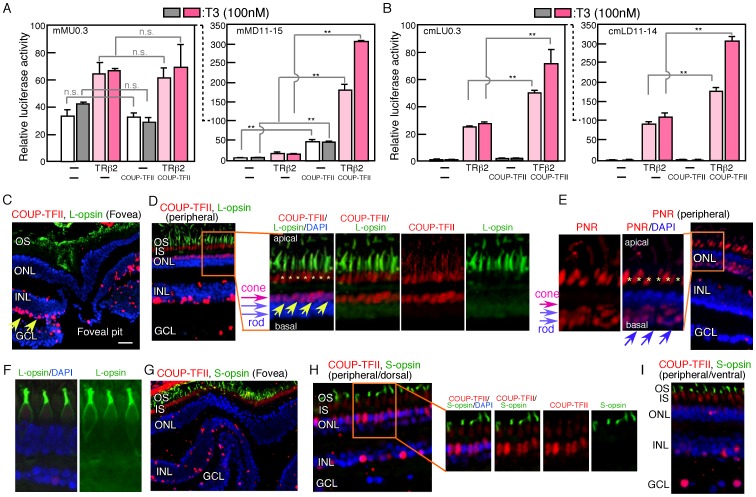
Promoter activities of mouse and common marmoset opsin genes, and expression of COUP-TFs in common marmoset retina. A. Luciferase constructs containing mouse M-opsin promoter (mRU0.3) or M-opsin exon/intron region (mRD11–14) were transfected into HEK293T cells with TRß2 expression plasmid or a control plasmid, and cultured for 12 hours, and T3 (100 nM) was added half of samples and cultured additional 16 hours. Luciferase activities were examined and expressed as relative values. B. Common marmoset long opsin promoter luciferase plasmid (cmLU0.3) or exon/intron construct (cmLD11–14) were transfected into HEK293T cells with TRß 2 expression plasmid and cultured and luciferase activities were analyzed as described for A. p value; **<0.01, n.s., (not significant), or actual value was calculated by Student’s t-test. C–F. Expression of COUP-TFII, L (long)-opsin (C, D), and PNR (E) in common marmoset retina. Common marmoset retina was fixed with PFA and frozen sectioned. Immunostaining was done, and photos of foveal pit region (C), peripheral region (D, E) are shown. The right panels in D and left panels in E are enlarged photos of a part indicated by orange squares of left (D) and right (E) photos. Arrows (yellow) in C and D indicate signals of L-opsin, and asterisks in D and E indicate non specific staining. Arrows (blue) in E indicate PNR signals. In outer nuclear layer, nucleus of cone aligned apical one layer (pink arrows), and basal nucleus are rods (blue double arrows) in D, and E. Scale bar in C, 100 µm. F indicates magnified photo of L-opsin immunostaining. G–I. Expression of COU-TFII and S (short)-opsin in common marmoset retina. Photos of foveal pit region (H), peripheral/dorsal (H), and peripheral/ventral (I) regions are shown. The right panels in H are enlarged photos of the part indicated by a yellow square in H. OS, outer segment; ONL, outer nuclear layer; INL, inner nuclear layer; GCL, ganglion cell layer; IS, inner segment.

Mouse M-opsin (Opn1mw) corresponds to human red opsin. We cloned the upstream region and intron 3–4 region of the gene and constructed luciferase reporter plasmids (Fig. 1D). The plasmids were transfected into HEK293T cells with or without TRß2. The luciferase activity of the plasmid that contained the 5′-region was enhanced by TRß2, while the addition of T3 did not enhance this activity (Fig. 4A). The lack of response of mMU0.3 to the addition of T3 was unexpected since previous work showed enhanced activity of a similar luciferase construct by T3 [23]. Differences in incubation time after transfection and in dose of T3 may have led to the differing results. Furthermore, the co-expression of COUP-TFII did not affect the luciferase activity either in the presence or absence of TRß2 (Fig. 4A). The luciferase activity of the plasmid that contained intron 3–4 was also slightly enhanced by TRß2, while the addition of T3 did not affect the luciferase activity (Fig. 4A). COUP-TFII did not suppress the luciferase activity, and even much higher luciferase activity of mMD11–15 was observed in the presence of COUP-TFII (Fig. 4A). COUP-TFII enhanced the luciferase activity, even in the absence of TRß2. However, the addition of T3 further enhanced the luciferase activities induced by TRß2 and COUP-TFII (Fig. 4A).

### Common Marmoset Red Opsin Gene Regulation

Since the observed opsin expression patterns in the mouse and human differed, we considered whether this reflected differences between rodents and primates. To clarify this issue, we used the common marmoset as a model system. The common marmoset has three different long wavelength (L) opsins, i.e., green (543 nm), yellow (556 nm), and red (563 nm), and these genes are the products of polymorphisms in an X-linked locus [24], [25]. We cloned the genomic DNA of the common marmoset L-opsin, comprised from −0.3 kb to TSS (transcription start site) and from +11 to 14 kb, and the corresponding luciferase reporter plasmids were constructed (Fig. 1E). Luciferase assay was measured in transfected HEK293T cells, and the activities driven by the upstream 0.3-kb region were enhanced by the expression of TRß2, while T3 did not enhance these activities (Fig. 4B). The addition of the downstream 11–14-kb region to the 0.3-kb promoter region resulted in much higher luciferase activities, as observed with the human and mouse red/M-opsins (Fig. 4B). However, the addition of T3 did not affect these activities (Fig. 4B). When COUP-TFII was co-expressed in the HEK293T cells, the luciferase activities were not affected in the absence of TRß2 but were enhanced when TRß2 was present in both cmLU0.3 and cmLD11–14 (Fig. 4B). Furthermore, the addition of T3 further increased the luciferase activities in both cases (Fig. 4B).

Since no suppressive effects of COUP-TFII on the common marmoset L opsin-luciferase fusion were observed, we next examined the expression patterns of COUP-TFII and L-opsin proteins in immunostained frozen sections of the common marmoset retina. As in the human retina, cone cells were found to be present at high density in the foveal region, whereas the expression of COUP-TFII was not clear in this region (Fig. 4C). In the peripheral region, COUP-TFII was expressed in the cone cells, in which L-opsin was also expressed (Fig. 4D, arrows in enlarged panel). Rod cells, labeled with PNR (photoreceptor-specific nuclear receptor protein, NR2E3) [26], did not show expression of COUP-TFII (Fig. 4E). The signals indicated by asterisks in the right panels of Figure 4D and E are non-specific signals. Enlarged photo of L-opsin staining (Fig. 4F) indicates L-opsin only in outer segments. Therefore, signals for L-opsin and COUP-TFII were not merged, but we conclude that they are expressed in the same cells, since COUP-TFII expressed in all cones in this region. S-opsin and COUP-TFII immunostainings are shown in Fig. 4G, 4H and 4I, and both COUP-TFII and S-opsin were expressed in cone cells in the peripheral region (Fig. 4H, I).

## Discussion

Previous studies have provided evidence that the expression patterns of cone opsins are unique among species, which suggests complex and specific mechanisms for generating these expression patterns. In the present study, we examined the activities of the promoters of the L/M-opsin genes of various species in relation to TRß2 and COUP-TFs. This work was initiated based on our hypothesis that the COUP-TFs differentially regulate opsin genes between the human and mouse. Our current results reveal highly complex regulation of opsin genes among species.

A role for thyroid hormone in cone pigments was first demonstrated using TRß2-knockout mice, which showed a loss of M cones and an increase in S-opsin [19]. Thereafter, several studies suggested essential roles for TRß2 in opsin expression in various species [27], [28]. In addition, a role for its ligand, thyroid hormone, was shown, whereby exogenous T3 inhibited S-opsin expression and increased M-opsin-positive cones [14]. We found that TRß2 enhanced luciferase activities through at least two different regions located in the 5′-UTR and intron 3–4 of the human red opsin gene. Interestingly, the luciferase activity induced by TRß2 through the 5′-UTR was not augmented by the addition of T3, while that of the intron 3–4 region was strongly increased by T3. Thyroid hormone receptors are known as ligand-dependent transcription factors, although there is a report describing ligand-independent transactivation by TRß2 [29]. That study showed that strong ligand-independent transactivation was observed with hormone response elements composed of direct repeats and everted repeats [29]. A search of the genomic sequence of the red opsin did not clarify whether this mechanism can be applied to the red opsin case. In the case of the common marmoset opsin, the addition of T3 did not affect the 5′-region and intron region, and in the case of the mouse opsin, expression of TRß2 only slightly upregulated the luciferase activities in the presence or absence of T3. Thus, regarding the mechanisms underlying this observation, we should consider not only the TRß2 binding site configuration but also other factor binding sites. Sequence alignment of the 5′ 0.3 kb region and downstream 8–11 kb region of mouse, human, and marmoset genomic sequences showed high similarity between the human and marmoset sequences, and high regional similarity between mouse and the other two species (Figure S1A, B). Number of potential binding sites for known transcription factors had been found in human sequence (Figure S1A, B), suggesting involvement of other factors to regulate opsin gene transcription. Since the transient luciferase analysis has limitation in the information it provides, precise examination of sequence homology combined with further analyses using other techniques such as EMSA, and footprinting will be valuable for confirming and extending our observations.

In fact, when COUP-TFII was co-expressed with TRß2, the mouse intron-dependent luciferase activity was synergistically activated, and in both the mouse and common marmoset, the opsin intron regions were responsible for stimulation by T3. Therefore, context-dependent synergy or changing the mode of activation of opsin gene by TRß2 may occur, although the underlying mechanisms remain obscure. Furthermore, we observed enhancing effects of COUP-TFII in certain situations. This was typically observed for the mouse intron 3–4 region. COUP-TF is known as a negative transcriptional regulator, although there are several papers showing transactivation by COUP-TF [30], [31]. Such a switch in the activity of COUP-TF may be regulated through interactions with other transcription factors.

The suppressive effects of COUP-TFII were stronger for the intron 3–4 region than for the 5′-region of the red opsin gene, which suggests that the mechanism of suppression mediated by COUP-TFII is different for these two regions. Suppression of the luciferase activity of the intron 3–4 region by COUP-TFII may not represent competition for the target sequence in the opsin gene between COUP-TFII and TRß2, as suggested by our experiment using COUP-TFDBD. Therefore, we assumed a direct interaction between TRß2 and COUP-TF and performed co-immunoprecipitation experiments to show interactions between TRß2 and COUP-TFs under different conditions. However, we did not obtain evidence of such an interaction (data not shown). Furthermore, ChIP analyses to identify target sequences using anti-TRß2 or anti-COUP-TFII antibodies were unsuccessful (data not shown). As an alternative way, we constructed many luciferase plasmids that contained further divisions of regions of the upstream promoter and intron region, so as to define more precisely the target sequences for TRß2 and COUP-TFII, but we did not get consistent results (data not shown). Therefore, we speculate that co-operation between multiple elements in the sequence of the opsin promoter generates the complex responses to TRß2 and COUP-TF. In the common marmoset, we found that both COUP-TFII and L/M-opsins were expressed in cone, and that luciferase activity was enhanced by COUP-TFII in combination with TRß2, thereby supporting the idea that COUP-TFII has positive effects on L/M-opsin expression in the common marmoset retina. In summary, our current results using different species reveal that the roles of COUP-TFs in opsin gene expression are spatially, temporally, and mechanically complex and are different across species.

## Supporting Information

Figure S1 **Multiple alignment of human, mouse and common marmoset opsin genes.** Multiple alignment in 5′ upstream (A) and intron 3–4 regions (B) of human, mouse and common marmoset opsin genes was done using VISTA tools (http://genome.lbl.gov/vista/index.shtml). The y axis of VISTA plot represents the conservation (%) between human/mouse or human/marmoset. Conserved regions above the level of 70% over 100 bp are shown in pink. Putative transcription binding sites (TFBS) of human sequence, analyzed using VISTA plots with a cut-off to minimize false.
(TIF)

Table S1 **Supporting information of experimental materials.**
(DOCX)
