# Supplementary figures and images for: Enhancer/Promoter Activities of the Long/Middle Wavelength-Sensitive Opsins of Vertebrates Mediated by Thyroid Hormone Receptor β2 and COUP-TFII
Source: PLoS One. 2013 Aug 23;8(8):e72065. doi: 10.1371/journal.pone.0072065 (PMC3751927; doi:10.1371/journal.pone.0072065)

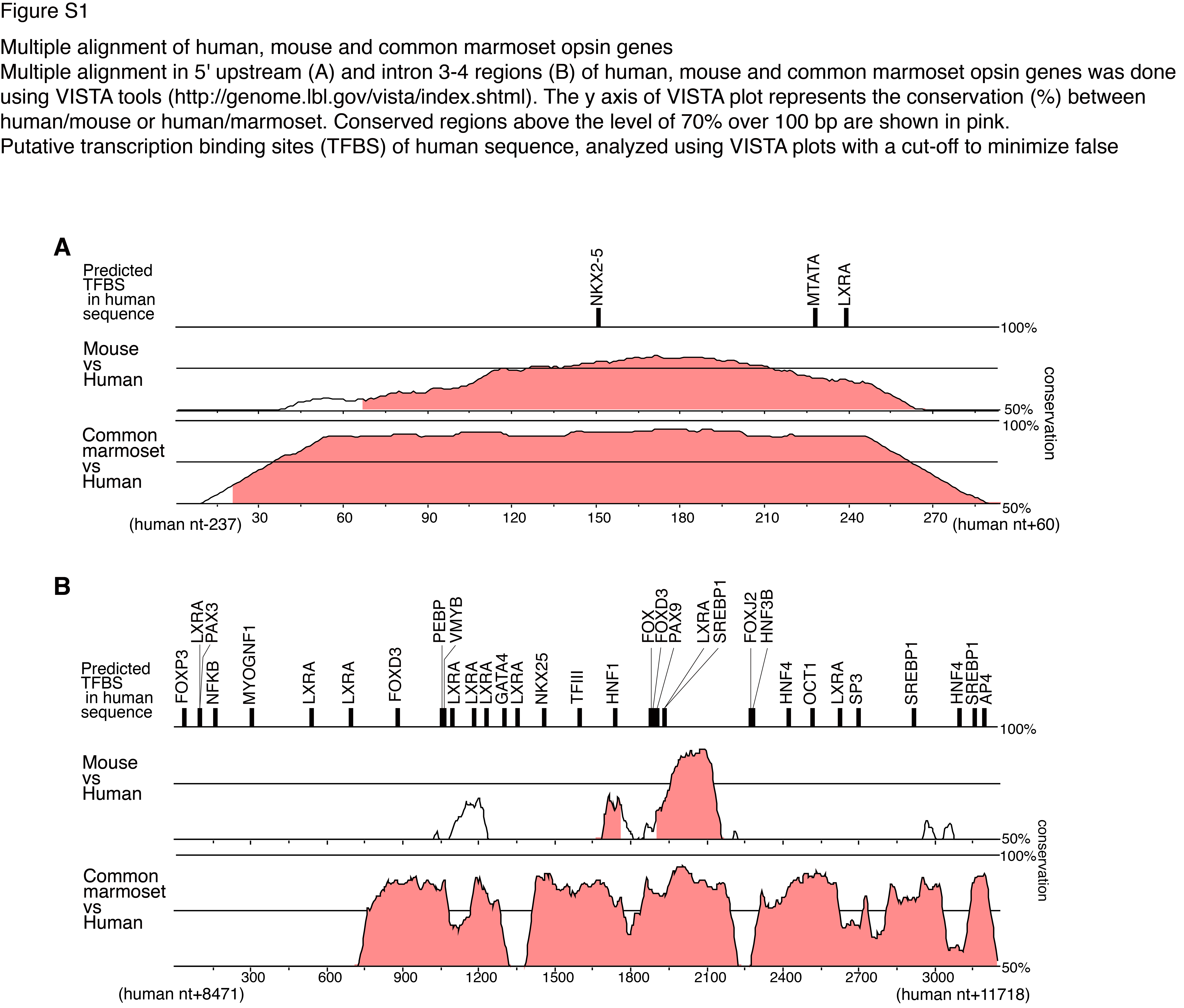

Supplement: Figure S1 — Multiple alignment of human, mouse and common marmoset opsin genes. Multiple alignment in 5′ upstream (A) and intron 3–4 regions (B) of human, mouse and common marmoset opsin genes was done using VISTA tools (http://genome.lbl.gov/vista/index.shtml). The y axis of VISTA plot represents the conservation (%) between human/mouse or human/marmoset. Conserved regions above the level of 70% over 100 bp are shown in pink. Putative transcription binding sites (TFBS) of human sequence, analyzed using VISTA plots with a cut-off to minimize false. (TIF) [file pone.0072065.s001.tif]
