# Supplementary material for: Enhancer/Promoter Activities of the Long/Middle Wavelength-Sensitive Opsins of Vertebrates Mediated by Thyroid Hormone Receptor β2 and COUP-TFII
Source: PLoS One. 2013 Aug 23;8(8):e72065. doi: 10.1371/journal.pone.0072065 (PMC3751927; doi:10.1371/journal.pone.0072065)
Supplement: Table S1 — Supporting information of experimental materials. (DOCX) [file pone.0072065.s002.docx]

Table S1

Supporting information of experimental materials

1. Primer list

1.1 Primer sequences for RT-PCR are as follows;

COUP-TFI 5’-aggtggagaagctcaaggcg-3’, 5’-tgaagctgctcccagacagt-3’

COUP-TFII 5’-gagcttcttcaagcgcag3’, 5’-taaaactgctgccggacagt-3’

G3PDH 5’-tgaccacagtccatgccatc3’, 5’-cataccaggaaatgagcttgac-3’

Red opsin 5’-ggtggtgatgatgatctttgcgt3’, 5’-cgataccgaggacacagatg-3’

Green opsin 5’-ggtggtgatggtcctggcatt3’, 5’-cgataccgaggacacagatg-3’

Blue opsin 5’-gctggacttacggcttgtca3’, 5’-ggccaatattgggtcctcag-3’

1.2 Primer sequences for RT-PCR for plasmid construction are as follows;

***For human gene construction***

***(constructs which are not listed were made by using existing restriction enzyme site)***

hRU0.1 5’-cccaagctttgatcccacaggccagtata-3’, 5’- ccatggctatggaaagccctg-3’

hRU0.2 5’-cccaagcttcaggagcaggggagcaag-3’, 5’-ccatggctatggaaagccctg-3’

hRU0.3 5’-cccaagcttagcggctcccacagtcac-3’, 5’-ccatggctatggaaagccctg-3’

hRU21 5’-ttggcgcgcggcctgggccaccctcacac-3’, 5’-cacgcgtaagagctcggta-3’

p55B9.1-11 5’-ctcgagatttagtccacagagtggcc-3’, 5’-aagcttagagttcagagccatcgtca-3’

***For mouse gene construction***

mMU0.3 5’-cccaagcttagtggcttccacaagcccta-3’, 5’-catgccatggctgtagaaaactg-3’

mMD11-15 5’-gaagatcttttgtgaagcttccat-3’, 5’-ccgctcgagaggctagaggccaaccac-3’

***For common marmoset gene construction***

cmLU0.3 5’-cccaagcttttcagctcccacaggcacc-3’, 5’-tgccatggctatggaaagccctg-3’

cmLD11-14 5’-cccggatcccgggccatgagcagagc-3’,5’-ccctctagagccaggctggccactctg-3’

***For COUP-TFII DBD mutant***

5'-ccggaattcatgtgcgtggtgtgcggggac-3',5'-atttgcggccgctcattcactctgtacagcttcccgtc-3'
